# Effect of Cryo-Treated Cutting Tool End Milling on Custom 450 Stainless Steel

**DOI:** 10.3390/ma16134744

**Published:** 2023-06-30

**Authors:** C. Devi, Siva Kumar Mahalingam, Robert Cep, Karel Kouril

**Affiliations:** 1 Department of Mechanical Engineering, Vel Tech Rangarajan Dr. Sagunthala R&D Institute of Science and Technology, Avadi 600062, India; 2 Department of Machining, Assembly and Engineering Metrology, Faculty of Mechanical Engineering, VSB—Technical University of Ostrava, 70800 Ostrava, Czech Republic; 3 Institute of Manufacturing Technology, Faculty of Mechanical Engineering, Brno University of Technology, Technicka 2896/2, 61669 Brno, Czech Republic

**Keywords:** end milling, cryogenic treatment, surface abrasion, chip anatomy, tool morphology, surface morphology, cutting force

## Abstract

Custom 450 stainless steel is the most desirable material across industries due to its widespread application in the aerospace, defense and marine industries. Stainless-steel materials are challenging to deal with and fall into the list of hard-to-process materials due to their low heat conduction coefficient and high mechanical properties. In this research work, end milling was carried out on Custom 450 stainless steel machined using TiAlN coated with tungsten carbide inserts that have been cryo-treated (CT) for 24 h (24 h) and 36 h (36 h), as well as untreated (UT) inserts. The inserts were evaluated in terms of feed force, feed rate and consistent depth of cut (ap) at various spindle speeds (S). Also examined were the tool morphology, chip anatomy and surface morphology of cryo-treated material compared to untreated inserts at various responses to cutting force (Fx, Fy, Fz), cutting temperature (Tc), vibration and surface abrasion. For inserts that have been cryo-treated for 36 h, the feed force (Fx) value was 44% and 5% less compared to inserts treated for 24 h and in UT inserts, respectively. Furthermore, for 24-h and 36-h CT inserts, feed force (Fx) was 12% and 20% less compared to a UT insert. Using 24-h cryo-treated inserts as opposed to UT inserts significantly reduced the surface roughness by 20%. Cutting inserts that have undergone cryogenic treatment have been observed to exhibit longer cutting tool life due to less wear and friction on the cutting edges.

## 1. Introduction

Stainless steels are frequently utilized in industries, including health, marine, defense and nuclear power plants as well as springs, nuts, bolts and screws due to their high strength and strong corrosion and oxidation resistance. This is because of their exceptional corrosion resistance, low heat conduction coefficient and good mechanical qualities. However, due to its numerous advantageous qualities, including high flexibility, high tensile strength, high fracture toughness and high work hardening rate, stainless-steel alloys are primarily employed [1]. Custom 450 stainless steel is used for aeronautical fittings, aerospace parts like bushings, shafts, valves and specific screws, as well as fuel tanks, exhaust components, high-temperature engine parts, structural parts and cabin components, landing gears and others. Custom 450 is a martensitic stainless steel grade with excellent corrosion resistance (up to roughly 650 °C) and may have its mechanical qualities greatly improved by heat treatment techniques [2]. Despite the fact that there are several pieces of research on the machinability of stainless steels in the literature, there is a dearth of publications about Custom 450 stainless steel. Moreover, information about end milling on Custom 450 stainless steel is not found in publications. This experiment is necessary due to the wide application of Custom 450 stainless steel.

Due to its high Ni and Cr content, stainless steel has exceptional hardness and machinability qualities. Hence, a very hard machining tool was needed. Tungsten carbide (WC-Co) is one of the most used materials in the industry, and cutting tools made of this material have a major impact on the effectiveness of machining processes and tooling costs. But the challenge with tungsten-coated carbide tools is that they wear out and reach failure rapidly [3,4]. This has the negative effects of poor surface quality, uneven tool wear, early tool failure and BUE (build-up edge) on the tool flank face and crater face during machining. BUE increases the frequency of tool wear and impairs the surface integrity of the work [5]. Working with hardened workpiece material increases the frequency of tool wear and damage, which has an impact on product correctness. Lowering the cutting temperature is the most feasible and successful approach to improving the machining efficiency of hard materials [6].

Cryogenic processing, also known as cryogenic treatment, was developed for the purpose of improving a material’s mechanical qualities. Through the microstructural alterations during treatment, it extends a cutting tool’s life. Additionally, it can enhance a material’s mechanical qualities from the core [7]. When cutting tool materials are subjected to cryogenic treatment, the austenite phase transforms into martensite [8,9]. The heat dissipation capacity is improved, the heating of the cutting edge is lowered and cutting tool wearing is decreased due to the increase in thermal conductivity [10]. Reddy et al. stated that the best method for reducing cutting force, extending tool life and improving tool wear resistance is to cryo-treat the cutting tools [11].

Cryogenic treatment is the greatest option for extending cutting tool life because of the lower tip temperature and better surface quality, which reduces wear on the cutting tool by 67% and produces a 20% better surface polish compared to untreated tools [12,13]. According to studies by Sola et al. [14], Gill et al. [15] and Dhokey et al. [16], cryogenic treatment considerably enhances material qualities including wear resistance, residual stresses, hardness, toughness and thermal conductivity. According to Düzce and others, Moore and Collins in 1993 and Sitki in 2015 investigated the cryo factors, such as the length of the cryo soaking period, the rate of cooling and the tempering procedure, which are all input parameters that affect how effectively cryogenic cutting tools perform and increase tool life and productivity [6,12,17].

When the coated cutting tool inserts undergo cryogenic treatment, they perform better than untreated cutting tools [18]. This treatment involves cooling the inserts to −196 °C and holding them there for a set period of time (for example, 24 h, 36 h and 48 h), and then bringing them to room temperature again slowly. Our tempering process followed cryogenic treatment and the results showed that a high rate of martensite transformation (α-Co to ε-Co) leads to improved mechanical properties. Sert et al. reported on the tempering process for 2 h at 200 °C. The transformation of α-Co decreases from 33.958% to 18.748% on WC-Co carbide [9]. Ozbek et al., in 2016, investigated the turning operations on AISI 304 stainless steel and stated that the tempering process releases internal stress. The result shows that this process improves wear resistance and increases hardness from 1709.8 to 1812.6 HV. This is due to the precipitation of fine alpha, beta and eta phase carbides and is proven by an improved carbide count from 2522 to 3330 [19]. According to Xun QIAO et al., Weng et al. and Kumar et al., the retained austenite is more stable when tempering temperatures are lower than 200 °C. The tempering of cryo-treated inserts involves increasing grain size and evenly distributing the number of carbide particles to enhance thermal conductivity and hardness [8,13,20]. According to research by Nirmal S. Kalsi et al. on the amount of post-tempering and cryogenic treatment performed on the carbide-cobalt insert tools used in turning operations, secondary carbides (W2 C and CO3 W3 C) are generated during the process, which improve the tool’s performance [21,22]. Korade et al. conducted several combinations of cryogenic treatment by increasing tempering temperature and increasing the number of tempering levels, which caused hardness to decrease and wear volume to increase. The machining performance and tool wear resistance during machining were both improved upon the cryogenic treatment of coated carbide inserts according to a literature survey [23]. Using a cryo-treated tungsten carbide cutting tool, Jadhav et al. performed turning operations on P20tool steel. The results revealed enhanced surface finishing and diminished cutting forces [2]. The turning insert’s microstructure and flank wear resistance both increased after cryogenic treatment, leading to higher machinability and less tool wear. In a turning operation on P25 conducted by Gill et al., according to their results, the cryogenic treatment improved the cutting tool life through an increase in flank wear resistance [15]. AISI316 stainless steel was the subject of a thorough investigation by Altan et al. in which the cryogenic treatment performance outcome demonstrated that the toughness was not compromised while the cutting tool’s wear resistance and hardness were increased [24]. Celik et al. demonstrated the increased wear resistance, cutting tool hardness and fracture toughness of cryo-treated cutting inserts when milling Ti-6Al-4 V titanium alloy that was machined using tungsten carbide [25]. Sivalingam et al. used a Ti-6Al-4 V alloy work material for milling using cryo-treated and untreated tungsten carbide inserts. Performance was looked into and, according to the study, cutting tools that have undergone cryogenic treatment guard against plastic deformation due to high spindle speeds. As a result, an enhanced surface roughness of 20–26% [26] can be attained. In a study by Gill et al. on hot-rolled steel in 2011, a study by Kývak et al. on M42 HSS drilling in 2015 and a study by Sahoo et al. in 2010, it was concluded that cryo-treated tungsten carbide inserts lengthen tool life at high spindle speeds. Surface finishing and tool life play key roles in the machining process. Coating and cryo treatment enhanced surface finishing and increased wear resistance [27,28,29].

This experiment is necessary since there are few publications on Custom 450 stainless steel. Despite the fact that there is much research in this area, cryogenically treated cutting inserts have received relatively little attention in the literature when used in drilling, milling and turning operations on different types of stainless steel. Moreover, end milling on Custom 450 stainless steel is not covered in any of the publications. Therefore, the current study is aimed at closing this research gap by examining the machining performance of Custom 450 stainless steel using a cyro-treated cutting tool with respect to the various responses to cutting force, cutting temperature and vibration. It is also aimed at examining the surface abrasion, surface morphology, chip anatomy and tool morphology of the workpiece. The results of this study may improve process optimization by providing a framework and enhancing our understanding of process behavior.

## 2. Materials and Methods

### 2.1. Material

**Figure 1:**
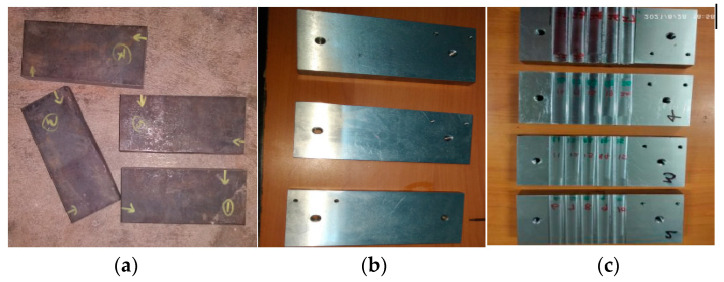
Preparation of workpiece: (**a**) before grinding (**b**) after grinding (**c**) after milling.

This study examines the workpiece material Custom 450 stainless steel prepared in the process of cutting and grinding, which is used to remove dust and rust on a workpiece and then obtain a glittering surface. The dimensions of 160 mm × 75 mm × 20 mm (4 numbers) were prepared as shown in Figure 1. The chemical composition and mechanical properties of Custom 450 stainless steel are shown in Table 1 and Table 2. In accordance with the ASTM-E8 standard [30], the specimens (workpieces) for the tensile test were made using a wire electro-discharged machine. The determined tensile qualities included yield strength, ultimate tensile strength and elongation. A Brinell hardness test was conducted using a 5 mm ball, 750 kg/load, and three values were observed and the average was taken into account. In accordance with the ASTM B294-10 standard [31], a rock well hardness tester was used to measure the hardness. In order to measure the hardness for the HRA scale, a major load of 573.4 N was applied after a minor load of 98.07 N to establish the sample. Three measurements of the hardness were taken to determine an average.

### 2.2. Experimental Setup

**Figure 2:**
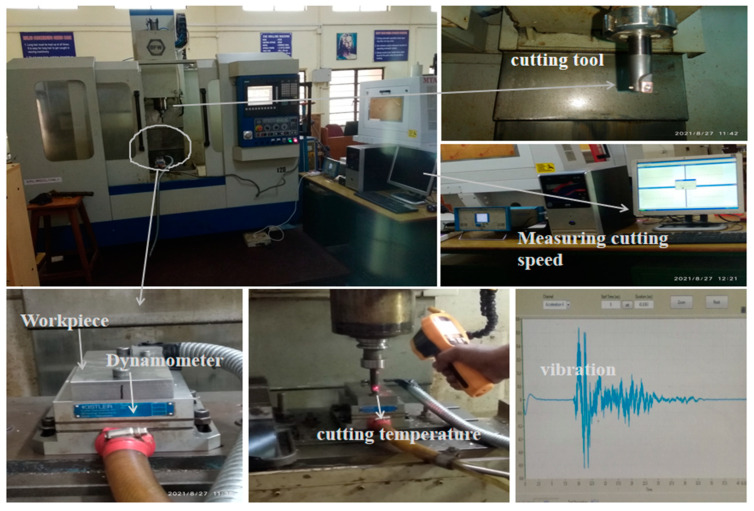
Experimental setup.

Custom 450 stainless steel was end-milled in a CNC vertical machining center at room temperature and in a dry machining environment. A TiAlN-coated tungsten carbide insert was chosen as the cutting tool insert and a normal indexable tool holder was used. The cutting insert was removed after the machining preset dimensions were reached, including a cutting length of 160 mm, a cutting width of 75 mm and a depth of cut of 1 mm in order to assess the tool wear performance. The complete experimental details are shown in Table 1. As the cutting parameters, the machining variables spindle speed (rpm), feed rate (mm/min) and depth of cut (mm) were used. The approach to experimentation for the current study is displayed in Table 2. Using a three-axis piezoelectric Kistler dynamometer (maximum forces up to 10 kN) to monitor the cutting forces, the data were collected in the system. The temperature was measured using an infrared thermometer of the noncontact IRX66 type (IR −50 to 1550 °C). This infrared light during the machining process moved along the machining cutting tool insert to measure in different locations and the average value was taken into account. In order to record the vibration signals during the milling process, a vibration sensor was attached to the spindle with the aid of a magnetic fixture. The KD10005LA acceleration sensor, a B&K data recorder and signal acquisition with analysis software V7.1 made up the majority of the vibration system. Signals from two directions were simultaneously measured by a vibration sensor. The X and Y axes of a sensor monitored the feed vibration and axial vibration, respectively, depending on where it was located on the tool. The end-milled surface gauged Mitutoyo’s Model SJ 210 gauge was used (accuracy of ±0.8 microns) to measure the end-milled workpiece’s surface roughness in three different locations and the average value was taken. The schematic view of the experimental setup is shown in Figure 2. The end milling chips and tool wear were observed using scanning electron microscopy (SEM) for further analysis.

### 2.3. Cryogenic Treatment

**Figure 3:**
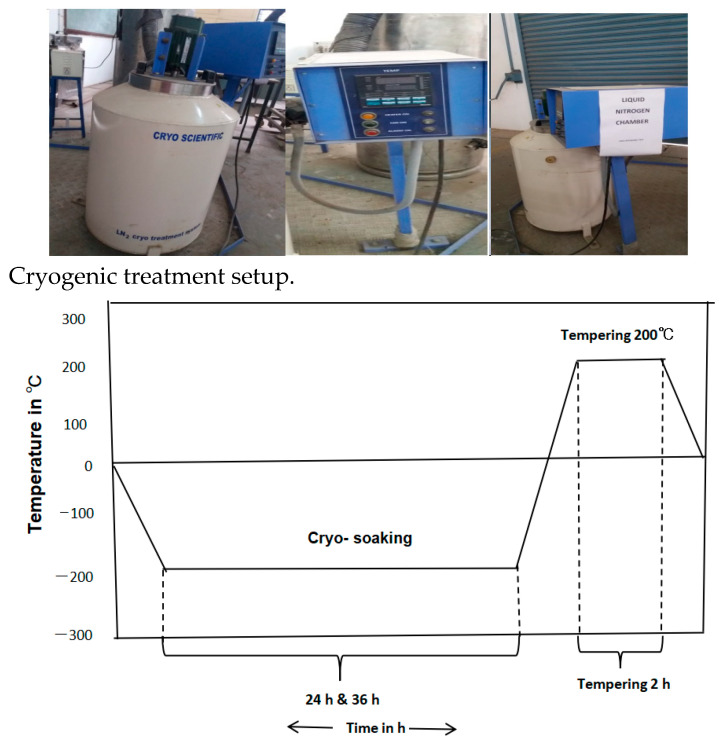
Cryogenic cycle of TiAlN-coated tungsten carbide insert.

The insert for the present work was coated with tungsten carbide. The detailed designation of the insert is shown in Table 2. In a custom-designed cryogenic chamber (KRYO 550-16), which can cool the sample down to a deep cryogenic temperature (−196 °C), the tungsten carbide inserts experienced cryogenic treatment. The cryogenic chamber was stuffed using gas produced in the chamber where the liquid nitrogen was originally stored. A computer controller was used to control how the cryogenic chamber interacted with an atomizer. Due to the controlled flow of liquid nitrogen inside the cryogenic chamber, a specific cooling rate was maintained for the temperature inside. Since the tungsten carbide insert was kept inside the sealed chamber and shielded from the liquid nitrogen, there was no risk of thermal shock harming it. Then, a 2 °C/min chilling process was used to gradually lower the cryogenic chamber’s temperature from room temperature (RT) to DCT (−196 °C). The temperature was maintained at this level (−196 °C) for 24 h and 36 h before being progressively increased to room temperature. As depicted in Figure 2, the cryo-treated tungsten insert was exposed to tempering cycles at 200 °C for 2 h to reduce tensions brought on by the cryogenic treatment (Figure 3).

## 3. Results and Discussion

### 3.1. Effect of Cryo-Treated Cutting Tool on Cutting Force, Vibration and Cutting Temperature

#### 3.1.1. Cutting Force

**Figure 4:**
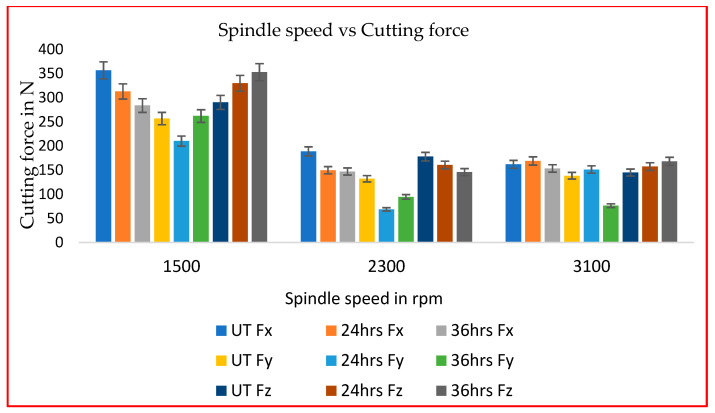
Spindle speed (rpm) vs. cutting force (F).

**Figure 5:**
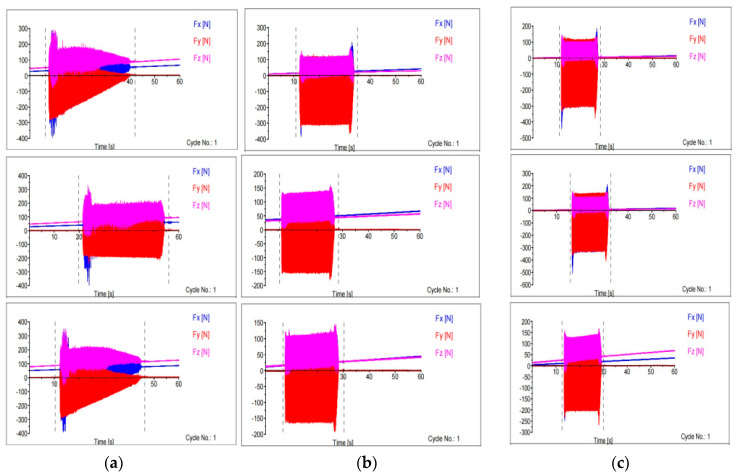
Cutting force at various speeds: (**a**) 1500 rpm (**b**) 2300 rpm (**c**) 3100 rpm.

The effects of the spindle speed on the feed force (Fx), normal force (Fy) and axial force (Fz) under all machining conditions are shown in Figure 4 with a constant feed rate of 0.1 mm/min. It was noted that the workpiece and cutting tools were initially quite rigid and the cutting force acquired maximum impact at lower cutting parameters. It caused milling machines to need greater torque power to shear the workpiece material at low spindle speeds (S) and feed rates (Vf). The feed force (Fx), was reduced by 12% and 20% for 24-h and 36-h cryo-treated cutting inserts, respectively, compared to UT. Normal force and axial force were slightly increased at low spindle speeds. After increasing the spindle speed from 1500 to 2300 rpm, the axial force (Fz) dropped by 9% when using 24-h CT and by 28% when using 36-h CT inserts, whereas the feed force (Fx) decreased by 20% when using 24-h CT and by 22% when using 36-h CT inserts. The normal force (Fy) decreased by 48% when using 24-h CT and by 28% when using 36-h CT inserts compared to UT cutting inserts. A lower cutting force was obtained in all directions by using cryo-treated cutting inserts. With the exception of the 36-h cryo-treated insert, which saw a 5% and 44% reduced cutting force compared to UT and 24-h CT inserts, respectively. Increasing the spindle speed typically caused a reduction in the cutting force. The impact of cryogenic treatment on the cutting force at a spindle speed of 3100 rpm, a feed rate of 0.1 mm per revolution and a cut depth of 0.5 mm is shown in Figure 5; the three forces are the Fx, Fy and Fz at various speeds.

#### 3.1.2. Cutting Temperature

**Figure 6:**
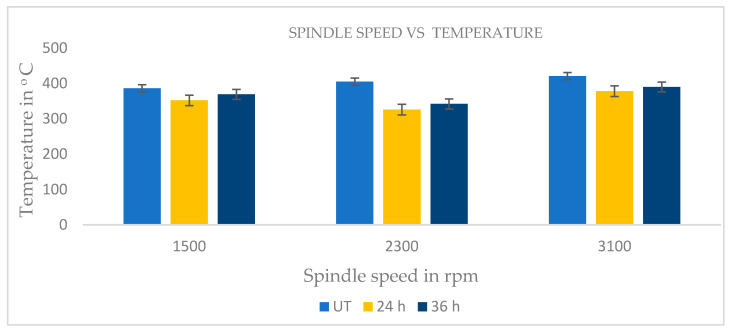
Spindle speed vs. cutting temperature.

The cutting temperature (Tc) in the workpiece–tool interface area (rake) was measured using a noncontact type IR thermometer with an accuracy of ±1.0 °C. By carefully focusing an IR ray on the cutting zone during milling and noting the greatest temperature reached, a tool–workpiece interface and heat were created. Due to friction between the tool and the workpiece, the cutting temperature was detected. Higher cutting temperatures result in less cutting force since the material may be deformed with less force. Due to the increasing shear stress, the workpiece became softer and required less cutting power to shear the material. Consequently, higher spindle speeds generated more heat in the cutting zone, shortened the tool–workpiece contact time and raised the shear plane angle [32]. When using 24-h cryo-treated cutting tool inserts, the cutting zone temperature was 5–6% less than the UT and 36-h cryo-treated cutting insert. This was proved by the cutting zone temperature at various spindle speeds and consistent feed rates (0.1 mm/min) as shown in Figure 6.

#### 3.1.3. Vibration

**Figure 7:**
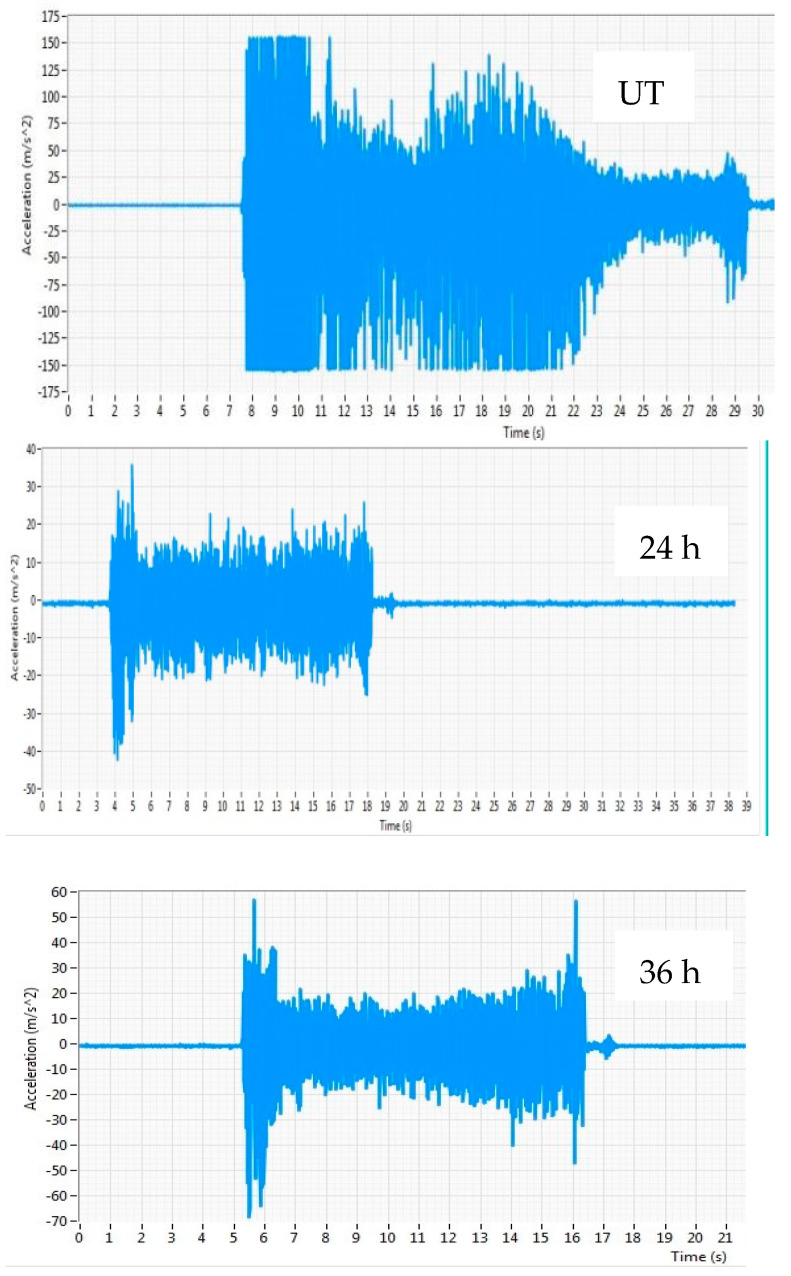
Cutting tool vibration at spindle speed of 3100 rpm.

Figure 7 shows the vibration of UT and cryo-treated cutting inserts. As the amount of friction between the workpiece and the UT cutting tool grew, the Tc also rose, leading to wear on the cutting edge. Using the 24-h cryo-treated cutting inserts resulted in lower friction due to the cutting zone temperature compared to the 36-h and UT inserts, as shown in Figure 6. The vibration is also one of the factors for tool wear. From the observed vibration, cryogenically treated inserts offer good strength and generate less vibration, resulting in superior surface finishes because of their exceptional rigidity and minimum tool wear even at higher spindle speeds, which the wear morphology SEM picture demonstrates. Cryo-treated cutting tool performance is better than that of the UT cutting inserts.

### 3.2. Tool Morphology

**Figure 8:**
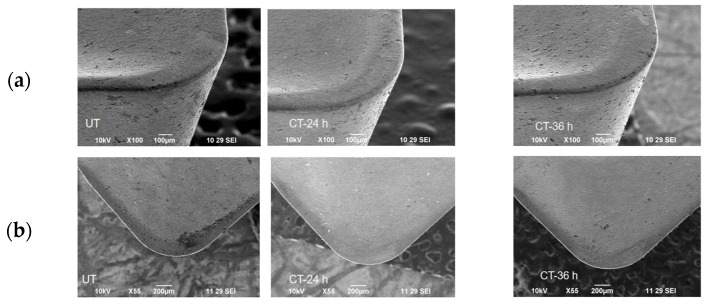
SEM image cutting insert at spindle speed of 3100 rpm, feed rate of 0.1 mm/tooth of UT, 24-h CT and 36-h CT, (**a**) flank face and (**b**) rake face.

Figure 8 shows the wear of tungsten carbide inserts as seen in SEM images. Cryo-treated inserts had substantially less flank chipping than untreated ones. Even at low cutting parameters, coating abrasion was detected in the rake face of the UT cutting inserts due to the UT tool inserts’ low wear resistance because the cutting zone temperature and friction both affected the UT inserts. Despite the fact that the material was substantially removed at high cutting parameters, the friction and cutting zone temperature had no impact on the cryo-treated inserts [33]. The preservation of the inserts’ sharpness through CT was employed to stop the degradation of cutting tool inserts. Treated cutting inserts’ rake and flank face showed no signs of wear.

In addition, the chips formed the build-up edge by sticking to the sharp cutting edges (BUE). As a result of work hardening over time, the generated BUE gradually became extremely hard and produced more vibrations and cutting forces. An abrupt plastic deformation at the tool edge resulted from this occurrence. Chips slipping over chip–tool interfaces caused craters because of the BUE of the cutting tool. Higher spindle speeds caused greater cutting temperatures in the cutting zone, which shortened tool life and caused increased tool wear. In addition, faster cutting speeds resulted in reduced tool-chip contact lengths, which concentrated cutting force towards the main cutting edge. The sharp edge became softer as Tc increased and the increasing Fx close to the cutting edge resulted in distortion and deflection [34,35].

### 3.3. Surface Abrasion

**Figure 9:**
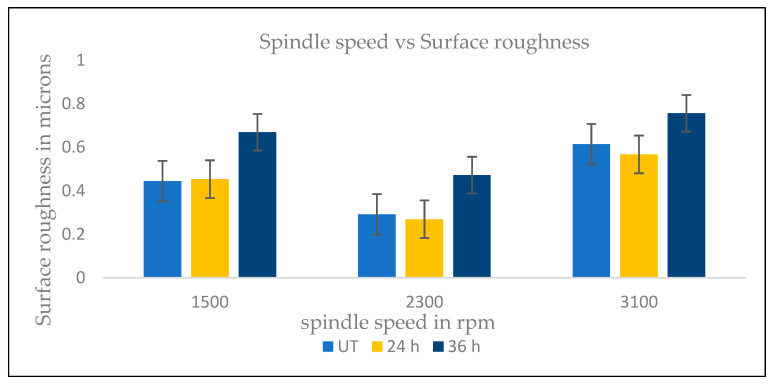
Surface abrasion in microns.

Figure 9 shows variations of (Ra) of end milling on stainless steel 450 machined by UT, 24-h and 36-h cryogenic-treated inserts. The end milling was conducted at feed rates (Vf) of 0.1 mm/min and spindle speeds (S) of 1500, 2300 and 3100 rpm and at a constant depth of cut of 0.5 mm. The 24-h cryo-treated cutting inserts performed better than the UT inserts. Cryo-treated cutting tools had less friction and a lower cutting zone temperature due to retaining their rigidity and sharpness during the cryogenic process. A 24-h cryo-treated cutting tool insert produced a better surface finishing of 0.269 microns while being fed at a low speed of 1500 rpm. By using 24-h cryo-treated inserts rather than the 36-h cryogenic-treated inserts and UT inserts, the surface roughness was reduced by 12%. At a high spindle speed of 3100 rpm, the cryo-treated cutting inserts improved the surface finishing by 20% compared to untreated cutting inserts. A 24-h cryogenic treatment enhanced the mechanical characteristics and preserved the hardness and attributes of the coating at a high temperature while creating less vibration than a standard insert. The untreated cutting inserts’ surface roughness was 28% higher due to increased friction and a higher cutting temperature. The flakes (chips) stuck to each other layer by layer due to the high temperature. This was due to the insert having undergone cryogenic treatment. This observation shows that cutting temperature has an impact on Ra.

### 3.4. Chip Anatomy

**Figure 10:**
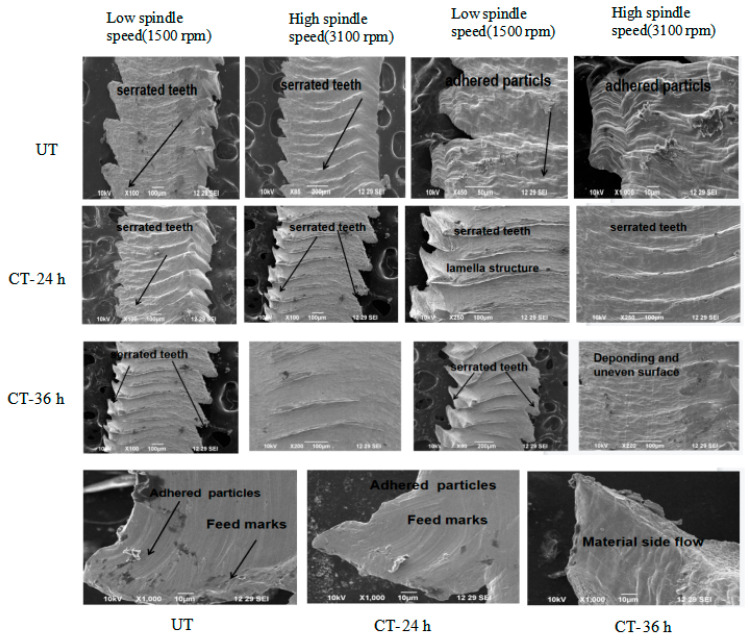
SEM image of chip anatomy.

Figure 10 shows the SEM image of chip anatomy after milling at different spindle speeds (1500, 2300 and 3100 rpm), a feed rate of 0.1 mm/min at a constant depth of cut of 0.5 mm. It is clear that the teeth were produced as serrated teeth after milling and have shrunk as a consequence of cryogenic treatment. When feed and cutting were maintained constantly, there appeared to be a rising trend in the chip segmentation frequency along with an increase in the cutting velocity. However, when machining was performed quickly, there was little time for chips to deform, making it impossible to effectively dissipate the cutting heat, which led to adiabatic shearing and then chip breakage. Vibration affects tool abrasion. Therefore, increased chip thickness in the UT cutting inserts may be seen as a result of vibration. The expansion of the shear plane area led to an increase in the chip thickness. As a result, as the spindle speed increased, the cutting force also increased, promoting the production of periodic chips and raising the chip thickness. An increase in Tc and high heat loads caused plastic deformation on the chips, as shown in Figure 10. Machining at high-temperature regions became easier due to thermal softening. A more significant number of serrated teeth can be seen on both sides of the untreated inserts at a high spindle speed (spindle speed (S) of 3100 rpm and feed rate of Vf 0.1 mm/min). When the material was removed at high temperatures, shear marks appeared and the chips were more deeply serrated because of the friction on the workpiece, and the chip particles adhered as shown in Figure 10. These tools had different chip lengths and twists in contrast to those under cryo-treated circumstances. The lengthier chips were a result of the intense friction that produced excessive heat during material cutting at a low feed rate and spindle speed, as well as the chips being stuck together [36,37].

Significant factors in chip morphology are the spindle speed and feed rate. Feed marks and saw teeth were observed from cryo-treated cutting tool inserts at high spindle speeds. Ridges appeared in white and dark lines in feed marks. The saw tooth length and chip separation frequency were the two most crucial chip characteristics. The chip separation frequency and saw tooth distance were inversely correlated [38]. There are still some ambiguous aspects regarding the mechanism of the formation of serrated chips despite their frequent occurrence during high-speed machining of ductile materials. By using a cryo-treated cutting tool insert, lamellar chips formed as shown in Figure 10, removing the material at the low heat generated. The lamellar chip formation process involved continuous and periodic chip formation. In this experiment, cryo-treated cutting inserts had no severe friction and less heat generation, identified by the small length of the curve type of chips. Due to the lesser amount of friction, wear did not occur on the cutting tool edges, which led to an increased tool life.

### 3.5. Surface Morphology

When machining a workpiece for a long time (increasing the number of passes), the feed marks became less sharp, resulting in the smearing of the working material. Smearing can occur during tool movement along the feed direction when the workpiece material flows to the side under crushing forces between the minor flank face and the machined surface. The following defects were observed from the machined surfaces.

**Figure 11:**
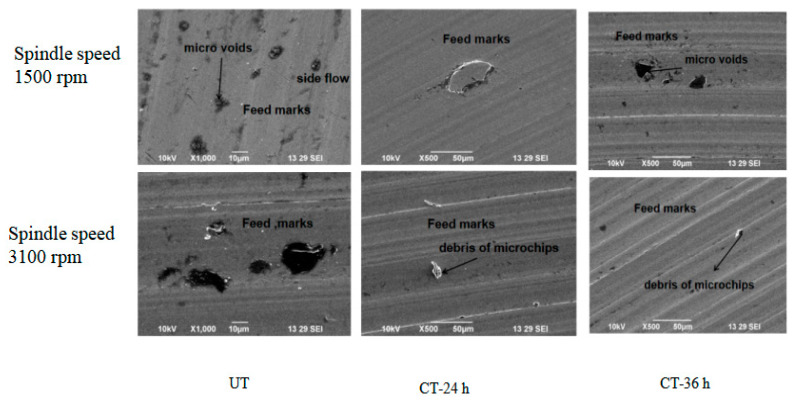
Surface morphology.

The workpiece material’s carbide particles and built-up edge (BUE) both aided in the creation of surface microvoids. The cutting tool, some of the BUE and the hard particles in the material could not deform at the plasticized layer because they were harder than the material of the workpiece. The hard particles cracked to release the strain. Following the removal of these particles, the chip and microvoids remained on the machined surface, as shown in Figure 11. Microvoids on the surface of a workpiece can adversely affect the subsequent mechanical properties of the component. Because UT and 36-h CT cutting tool inserts are less rigid, they produced microvoids. Due to their lower rigidity, they did not remove material properly at a low spindle speed and the chips stuck to the surrounding of the microvoids [39]. According to Figure 10, the 24-h cryo-treated cutting tool inserts led to better machining performance. Since the cutting tool was sharp, it removed chip material from the workpiece and did not generate more heat during the machining process. In order to make this observation, the Fx and Ra were measured. There were feed marks visible in every sample that was machined. In all the machined samples, feed marks were observed. The spindle speed and flank wear had a considerable impact on the surface feed markings on machined workpieces. It was discovered that feed marks on the machined surface were brought about by tool rotation and tool motion, which together mapped the lay pattern. Furthermore, there was a great deal of microchip debris visible. The flaw was brought about due to cracked chips being scattered throughout the cutting surface. Chip materials were deposited on the surface of the workpiece as a result of the high heat produced by the UT cutting tool.

High local cutting temperatures generated by fast cutting can cause pitting corrosion on the torn surface. Tool–workpiece interfaces formed melting layers and surface layers underwent phase transformation, leaving oxides and metal debris adherent to the machined surface at a high spindle speed (3100 rpm, UT), as shown in Figure 10. The way a burr forms during machining can be used to determine the type of burr and the mechanism of its production. A material will swell at the sides when compressed until it experiences plastic deformation. A 36-h cryo-treated and UT cutting tool used at the same spindle speed after machining a workpiece produced high depths of feed marks, debris and microvoids compared to a 24-h cryo-treated cutting tool [39]. Figure 10 shows that the 24-h cryo-treated cutting tool performed better than the 36-h cryo-treated inserts.

## 4. Conclusions

The aim of this research was to analyze the milling operations of Custom 450 stainless steel pieces using TiAlN-coated tungsten carbide inserts in UT and cryogenically treated (24-h CT and 36-h CT) conditions. Evaluations were performed on the vibration, chip anatomy, Fx, tool morphology, Ra and other output reactions.

- When UT and CT were compared at low spindle speeds, the feed force decreased by 12% and 20%, respectively, by cryo-treating the cutting insert for 24 h and 36 h. The cutting force of the 36-h cryo-treated inserts reduced to 5% and 44% compared to the UT and 24-h cryo-treated inserts, respectively. Due to a lower Tc compared to the 36-h and UT inserts, friction was reduced when the cutting inserts were employed after being cryo-treated for 24 h. Cryogenically treated inserts had good stiffness and less tool wear, even at higher spindle speeds.
- The friction or temperature of the cutting zone did not affect the cryo-treated inserts. Cryogenic treatment was used to stop the degeneration of cutting tool inserts because it maintains the inserts’ sharpness. On the treated cutting inserts, rake and flank face wear were undetectable.
- Cryo-treated cutting tool inserts that were fed at a low speed for 24 h at a rate of 1500 rpm resulted in a superior surface polish of 0.269 microns. The surface polish generated by cryo-treated cutting inserts was 20% better than that by untreated cutting inserts at a high spindle speed of 3100 rpm. A 24-h cryo-treated insert improved the cutting tool inserts’ mechanical properties while maintaining the coating’s quality and toughness at high temperatures.
- The results of this experiment demonstrate that cryo-treated cutting inserts produce less heat and have low levels of friction. The cutting tool edges do not wear out as quickly due to the reduced friction, which may result in an increase in the tool’s life.
- When employed at the same spindle speed, a 36-h cryo-treated cutting tool and UT tool left behind a workpiece with more feed marks, debris and microvoids than a 24-h treated cutting tool produced.

## Figures and Tables

**Table 1:** Custom 450 stainless steel chemical structure and properties.

Property	Value
Chemical composition (content Wt%)	C-0.029; Si-0.387; Mn-0.528, P-0.015, S-0.002; Cr-14.67, Mo-0.498; Ni-6.346; Cu-1.856, Nb-0.655; Fe-remaining
Ultimate Tensile Strength (MPa)	1020
Yield Strength (MPa)	939
Elongation (%) (50 mm gauge length)	17.5
Brinell hardness test (HB)	309
Rockwell hardness (HRC)	32.5

**Table 2:** Machining parameters.

Machine Tool	CNC Milling Machining
Workpiece material	Custom 450 stainless steel
Cutting tool insert	TiAlN-coated tungsten carbide, APMT1135PDR YBG205
Cutting tool insert thickness	3.5 mm
Tool holder	Diameter: 16 mm, length:150 mm
Cutting environment	UT, CT 24 h and CT 36 h (dry)
Spindle speed	1500 rpm,2300 rpm,3100 rpm
Feed rate (f)	0.1 mm/min
Depth of cut	0.5 mm

## Data Availability

The data presented in this study are available upon request through email to the corresponding author.
